# Extracting knowledge networks from plant scientific literature: potato tuber flesh color as an exemplary trait

**DOI:** 10.1186/s12870-021-02943-5

**Published:** 2021-04-24

**Authors:** Gurnoor Singh, Evangelia A. Papoutsoglou, Frederique Keijts-Lalleman, Bilyana Vencheva, Mark Rice, Richard G.F. Visser, Christian W.B. Bachem, Richard Finkers

**Affiliations:** 1 Plant Breeding, Wageningen University & Research, PO Box 386, Wageningen, 6700AJ The Netherlands; 2 IBM Netherlands, Amsterdam, The Netherlands

**Keywords:** NLP, Plant science literature, IBM Watson, Text mining, Relationship extraction, Knowledge networks

## Abstract

**Background:**

Scientific literature carries a wealth of information crucial for research, but only a fraction of it is present as structured information in databases and therefore can be analyzed using traditional data analysis tools. Natural language processing (NLP) is often and successfully employed to support humans by distilling relevant information from large corpora of free text and structuring it in a way that lends itself to further computational analyses. For this pilot, we developed a pipeline that uses NLP on biological literature to produce knowledge networks. We focused on the flesh color of potato, a well-studied trait with known associations, and we investigated whether these knowledge networks can assist us in formulating new hypotheses on the underlying biological processes.

**Results:**

We trained an NLP model based on a manually annotated corpus of 34 full-text potato articles, to recognize relevant biological entities and relationships between them in text (genes, proteins, metabolites and traits). This model detected the number of biological entities with a precision of 97.65% and a recall of 88.91% on the training set. We conducted a time series analysis on 4023 PubMed abstract of plant genetics-based articles which focus on 4 major Solanaceous crops (tomato, potato, eggplant and capsicum), to determine that the networks contained both previously known and contemporaneously unknown leads to subsequently discovered biological phenomena relating to flesh color. A novel time-based analysis of these networks indicates a connection between our trait and a candidate gene (zeaxanthin epoxidase) already two years prior to explicit statements of that connection in the literature.

**Conclusions:**

Our time-based analysis indicates that network-assisted hypothesis generation shows promise for knowledge discovery, data integration and hypothesis generation in scientific research.

**Supplementary Information:**

The online version contains supplementary material available at (10.1186/s12870-021-02943-5).

## Background

Scientific publications accumulate knowledge and developments in any field of research. One of the most important tasks in a researcher’s work and career is keeping up to date with the ever-increasing volume of scientific literature, placing new outputs into context, and investigating the implications in their field. However, as the number of scientific publications is growing at an exponential rate, there is a need to use artificial intelligence to enable a machine to read, extract, and analyze the information in textual sources.

Potato (*Solanum tuberosum L.*) is one of the most important staple crops for human nutrition. In addition to its culinary versatility, potato is a cost-effective product and plays a major role in meeting the ever-increasing food demands of the world. Its tubers are a good source of starch, proteins, and vitamins [1]. Different potato genotypes produce tubers of different properties, like shape, size, color, starch content, and nutritional value.

One of the most extensively studied traits in potato is tuber flesh color. Potato tubers can have a wide range of colors, from orange to white and purple. Carotenoids are considered to be the primary determinant of tuber flesh color [2]. Carotenoids play essential roles in photosynthesis, while in non-photosynthetic tissues, they exert a broad range of functions acting as pigments, antioxidants, and precursors of signaling molecules, including volatiles [3]. Previous studies have shown that zeaxanthin and its precursor beta-carotene are major determinants of tuber flesh color [4, 5]. In recent years, several candidate genes like beta-carotene hydroxylase (BCH/CHY2) and zeaxanthin epoxidase (ZEP) have been found to relate to tuber flesh color. BCH/CHY2 are the genes related to the production of beta-carotene while ZEP is considered responsible for the accumulation of zeaxanthin [6]. Although high levels of beta-carotene accumulating in transgenic tubes are not observed in tetraploid potato cultivars [5], alleles contributing to orange flesh have been observed at a low allele frequency in the potato cultivars [4]. This suggests that breeding has selected for the light colored alleles.

Scientific evidence for the association of tuber flesh color with genetic and molecular entities is found in the scientific literature or biological databases. For example, Acharjee et al. previously published networks of experimentally found biological entities that relate to tuber flesh color in the years 2011 and 2016 [6, 7]. In this research, we automate the process of extracting knowledge of molecular entities (genes/proteins/metabolites) that influence changes in tuber flesh color from scientific publications.

Compared to structured information (as in databases), textual information is huge, noisy, and redundant. Artificial intelligence can help automate the processing of textual information and the discovery of new knowledge. Natural Language Processing (NLP) is a field of artificial intelligence that focuses on enabling machines to understand and analyze (unstructured) data in the form of text [8]. Despite the availability of various data repositories for plant research, a wealth of information currently remains buried within the scientific literature. Hence, information extraction via NLP is of growing interest and importance. NLP can render scientific texts computationally accessible, support information extraction, knowledge network (KN) construction and hypothesis generation.

In the past years, many NLP based research studies have been conducted on the literature from molecular biology [9, 10]. These focused primarily on rule-based named entity recognition (NER) i.e. identifying and annotating biological entities such as genes or proteins [11, 12], metabolites [13, 14], traits [15], QTLs [16], diseases [17], and drugs [18] in literature. A few NLP studies paid attention to extracting associations (relationships and events) between these biological entities, using NER systems under the hood [12, 19, 20]. Automated approaches to mining knowledge concerning the association of an entity to its phenotypes are required to further advance the field of precision breeding [21]. Rule-based NLP is more widely used in mining knowledge from biological context than machine learning-based NLP [22, 23]. However, construction and formalization of rules is a complex task in rule-based NLP. Often the rule-based NLP user tends to overfit the rules to the training set, which affects performance in the test set. Dictionaries and ontologies are used as building blocks in rule-based NLP. In supervised machine learning-based NLP, on the other hand, a domain specialist annotates the training set of documents manually. These manually annotated documents, supported by dictionaries and ontologies, are used by an algorithm to produce context-specific rules. Finally, these rules are used to perform NLP on the unannotated test set.

In this research, we investigated whether the latent knowledge in scientific literature can be harnessed with NLP, and if new leads for gene-trait associations can be highlighted for hypothesis generation in a timely manner. However, our contribution is not in the domain of NLP, but rather in uncovering its potential. We chose to focus on the flesh color of potato tubers, an agronomically important trait with known associations. This enabled us to compare the relationships that we distilled from the literature with established facts, serving as a metric for the performance of our pipeline. It was necessary to validate more secondary hypotheses before we could focus on the time dimension of this question, namely 1) whether the NLP model is able to extract the expected relationships from the free text in literature; and 2) whether abstracts alone can act as high-certainty, information-dense proxies for their corresponding articles.

Our pipeline started with the NLP model, which was customized based on domain-relevant literature to find biological entities (genes, proteins, metabolites, and traits) and general relationships between them. We chose to use the commercial IBM (International Business Machines Corporation) Watson software suite, as it has been previously used to successfully mine knowledge from large corpora of texts available online [24, 25]. Watson Knowledge Studio is a proprietary cloud-based application to train an NLP model based on the context and linguistic nuances of a specific literature domain. In addition to annotating entities of interest in a given text (named entity recognition), Watson also performs relationship extraction; that is, labeling the connections between the detected entities of interest. The relationships extracted by Watson were used to build KNs. After a normalization step, we were able to integrate these, and produce visualizations of the distilled knowledge from a set of texts.

We composed a primary corpus of 34 selected articles, mainly concerning potato flesh color, which we used to train our NLP model. Later we deployed it on a subset of these 34 (abstracts only) and a broader-spectrum corpus comprising 4023 PubMed abstracts, published from 2000 to 2016. For the former, we compared the nodes and the edges of the networks to test our secondary hypotheses. For the latter, we also performed a time-based analysis, tracking the closeness of our trait of interest to other relevant entities, marking the time points where significant developments occurred, to evaluate whether this approach is indeed helpful for research. This time analysis and the results derived from it is our major contribution.

This proof of concept (although limited in size) is an example of how literature mining can help plant scientists obtain a clearer “big picture” about specific areas in their field of expertise. Elusive findings in the expanding body of literature could come to light, be automatically organized into KNs, and ultimately help accelerate research in a process with little human intervention.

## Results

First, to confirm that our domain-specific NLP model performed as intended and extracted knowledge networks (KNs) with the focus on tuber flesh color from scientific literature, we deployed it on 2 different corpora, i.e. the training set with full-text articles and the test set with PubMed abstracts only. This was followed by a time analysis on the test set, to investigate whether the knowledge in these KNs could really be used in the way we envision, to generate new hypotheses.

### Case 1: analysis of training corpus (full-text articles)

**Fig. 1:**
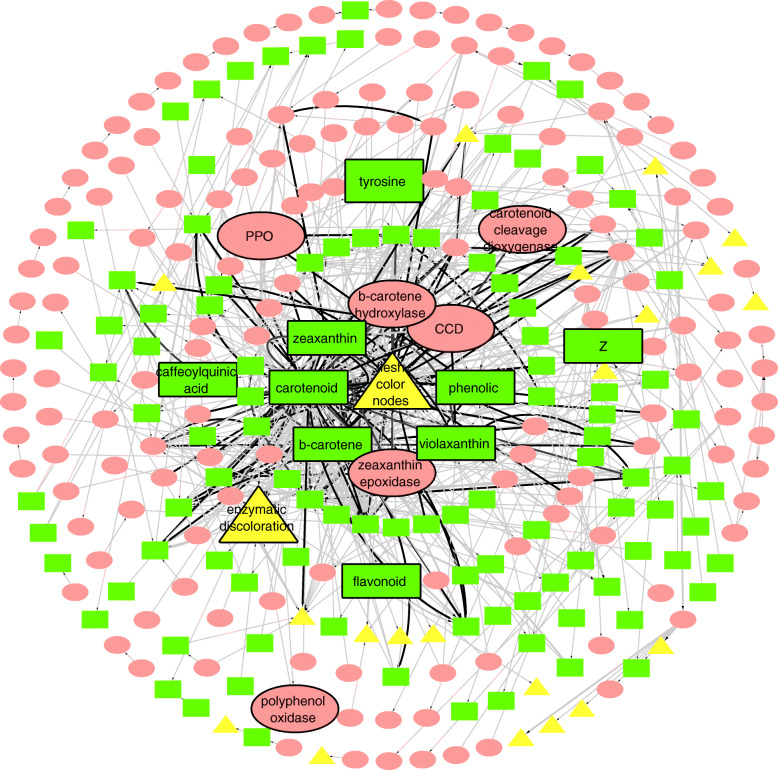
A KN representing knowledge triples found in the training set of 34 full articles. Yellow nodes refer to a trait entity, red nodes refer to gene/protein entities, and green nodes represent the metabolite entities. The centroid of this network is tuber flesh color. Nodes with bold outlines indicate that these entities have an experimentally proved association with tuber flesh color (trait of interest). This experimental evidence of these entities with tuber flesh color is reported in the articles [6, 7]. The color of edges reflects the document frequency (weight) of a relationship. Grey edges appear only in one document, whereas black edges appear in more than one. The nodes are organized in circles around the trait of interest. The nodes in the innermost circle (circle 1) and the 2nd innermost circle (circle 2) are 1st order neighbours of the flesh color node; the nodes in circles 3, 4 and 5 are 2nd order neighbours of it; the nodes in circle 6 are 3rd order neighbours; nodes in circle 7 (the outermost one) are higher order neighbours of the flesh color node, or not connected to it at all

We built a KN on the training set of 34 articles, with a total of 293 nodes and 551 unique edges. Out of these 293 nodes, there are a total of 159 genes/proteins, 112 metabolites and 22 traits (Fig. 1). Carotenoids (an entity of the type metabolites) was the primary centroid of this network having 76 first-order neighbors. To evaluate the nodes and connections of this KN, we analyzed the overall structure based on the currently known experimental knowledge of tuber flesh color. Our KN contains scientifically credible links between nodes and the trait of interest, tuber flesh color. Most genes/proteins and metabolite entries in this network are part of the carotenoid biosynthesis pathway, which includes beta-carotene biosynthesis, xanthophyll cycle, abscisic acid biosynthesis, lutein biosynthesis, etc.

The trait under study, tuber flesh color, has 38 first-order neighbors, comprising 11 genes/proteins and 27 metabolites (the Cytoscape network can be found at [26]). These genes/proteins and metabolites are also listed in Table 1. Previously conducted research studies have found that ZEP and BCH/CHY are associated with white, yellow and orange flesh color. AN1, a gene responsible for the production of anthocyanin, is associated with purple flesh color. All these genes occur as direct neighbors of tuber flesh color in our network.

**Table 1:** Sets representing first order (direct) neighbors of flesh color nodes. Set A represents first-order neighbors of tuber flesh color nodes found in full-text articles. Set B represents first-order neighbors of tuber flesh color nodes found in abstracts of articles of the training set. The difference between these sets (SET A - SET B) represents all entities that are first-order neighbors of tuber flesh color in full-text articles, but not in abstracts alone

Set A	Set B	Set A - Set B
AN1	anthocyanin	AN1
anthocyanidin	ascorbic acid	anthocyanidin
anthocyanin	b-carotene	carotene hydroxylase
ascorbic acid	b-carotene hydroxylase	cyanidin
b-carotene	bHLH	epoxides
b-carotene hydroxylase	caffeic acid	essential amino acids
bHLH	carotenoid	glycosides
caffeic acid	CCD	lutein
carotene hydroxylase	chlorogenic acid	lutein-5,6-epoxide
carotenoid	CHY	malvidin
CCD	Or	nonepoxide
chlorogenic acid	phenolic	pelargonidin
CHY	TP	peonidin
cyanidin	tuberigen activation complex	petunidin
epoxides	xanthophyll	Pf
essential amino acids	zeaxanthin	phenolic acid
glycosides	zeaxanthin epoxidase	phytoene synthase
lutein		polyphenol
lutein-5,6-epoxide		recessiveZEP
malvidin		violaxanthin
nonepoxide		violaxanthin-like carotenoid
Or		
pelargonidin		
peonidin		
petunidin		
Pf		
phenolic		
phenolic acid		
phytoene synthase		
polyphenol		
recessiveZEP		
TP		
tuberigen activation complex		
violaxanthin		
violaxanthin-like carotenoid		
xanthophyll		
zeaxanthin		
zeaxanthin epoxidase		

Our NLP model retrieved the entities in the training set with a precision of 97.65%, a recall of 88.91% and an F1 score of 93.07%. Supplementary File 1 presents a confusion matrix showing the total number of entities per document, number of true positives (TP), number of false negatives (FN) and number of false positives (FP). Precision and recall were calculated as TP / (TP + FP) and TP / (TP + FN) respectively.

Additionally, to compare the difference in volume and quality of information extracted from abstracts vs. full-text versions of articles, our NLP model was applied separately on only the abstracts of the training corpus.

**Fig. 2:**
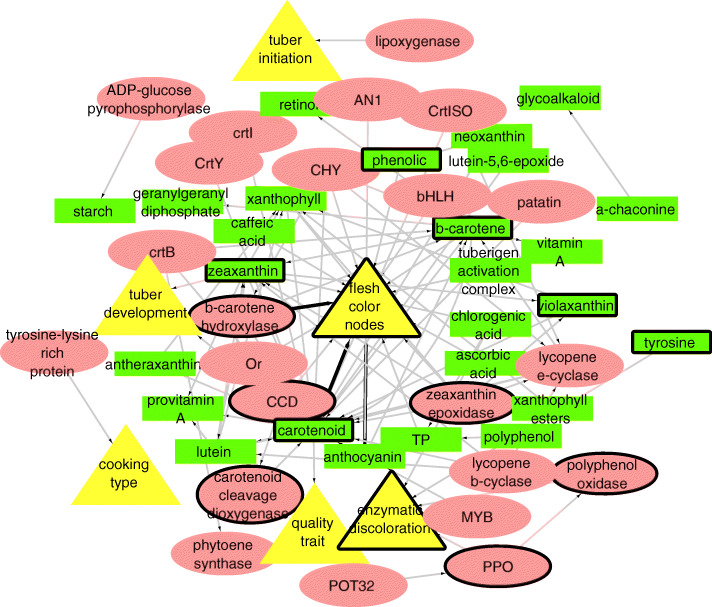
A KN representing knowledge triples found in the training set of 34 articles, abstracts only. Yellow nodes refer to a trait entity, red nodes refer to gene/protein entities, and green nodes represent the metabolite entities. The centroid of this network is tuber flesh color. Nodes with bold outlines indicate that these entities have an experimentally proved association with tuber flesh color (trait of interest). This experimental evidence of these entities with tuber flesh color is reported in the articles [6, 7]. The color of edges reflects the document frequency (weight) of a relationship. Grey edges appear only in one document, whereas black edges appear in more than one. The nodes are organized in circles around the trait of interest. The nodes in the innermost circle (circle 1) are 1st order neighbours of the flesh color node; the nodes in circle 2 are 2nd order neighbours of it; the nodes in circle 3 (the outermost one) are 3rd (or higher) order neighbours of the flesh color node, or not connected to it at all

This highlighted a quantitative difference between these two representations of a scientific article. We hypothesized that the abstract would concretely and concisely present the core outputs of a publication, whereas the “Introduction” section would mainly recapitulate established theories and relevant biological connections but without contributing new knowledge. Finally, the “Results” and “Discussion” sections would combine, in greater detail, the significant contributions of the article, and make further suggestions for future experimentation. We found supporting evidence for this hypothesis, as the abstract-only network still includes the entities experimentally shown to be most important for tuber flesh color. In Sets A and B, Table 1 lists the direct neighbors of tuber flesh color node in the KNs of full text representation (Fig. 1) and abstracts only (Fig. 2).

The difference between these two sets (Table 1; Set A - Set B) is also shown. These 20 entities occur as direct neighbors of flesh color in the full-text KN, but not in the abstract-only KN. Of these 20 entities, 6 (AN1, lutein, lutein-5,6-epoxide, polyphenol, phytoene synthase, violaxanthin) are still present in the KN of abstracts (Fig. 2), even though they are not direct neighbors, but rather second-order neighbors of tuber flesh color and first-order neighbors of carotenoids, BCH, or ZEP. Furthermore, recessive ZEP is also represented in the abstract-only KN. Since the recessive allelic variant of ZEP is similar to the dominant one, these nodes are not represented as separate entities. The same applies to other aspects of gene/protein characteristics, such as chemical isomers and trait measures, which we grouped together with the main entity to reduce fragmentation in our KNs. The remaining 12 entities (nonepoxide, peonidin, anthocyanidin, petunidin, pelargonidin, cyanidin, pf, malvidin, epoxides, glycosides) are not represented in the abstract-only KN. These entities are associated with key metabolites causing changes in flesh color. However, they do not influence the trait directly. Hence, our results illustrate that the most important nodes in the full-text network are still present in the reduced abstract-only network.

### Case 2: analysis of testing corpus (PubMed abstracts)

**Fig. 3:**
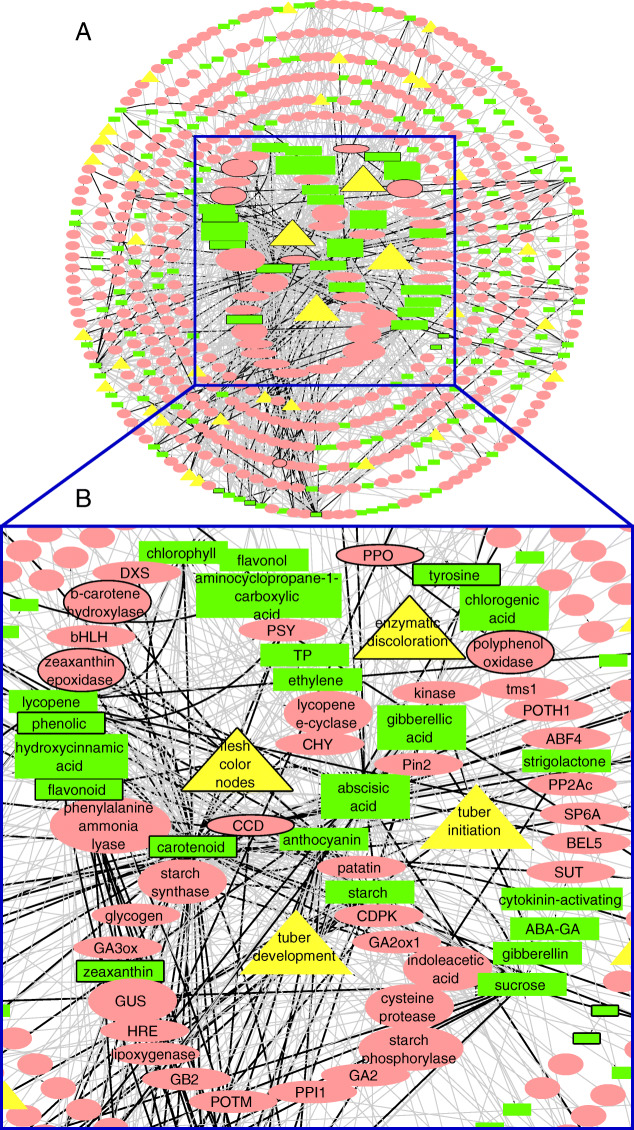
A KN representing knowledge triples found in the test set of 4023 PubMed articles. Yellow nodes refer to a trait entity, red nodes refer to gene entities, and green nodes represent the metabolite entities. **a** complete zoomed-out knowledge network **b** zoomed-in snapshot of the knowledge network focusing on tuber flesh color and additional traits with their respective biological associations. The color of edges reflects the document frequency (weight) of a relationship. Grey edges appear only in one document, whereas black edges appear in more than one. The nodes are organized in circles around traits of interest. Each of those traits has its 1st order neighbours in a circle around it (circle 1). The remaining nodes are organised with respect to the main trait of interest (flesh color nodes). The nodes in circles 2, 3 and 4 (counting from the center) are 2nd order neighbours of the flesh color node; the nodes in circles 5 and 6 are 3rd order neighbours of it; the nodes in circle 7, 8 (the outermost ones) are 4th (or higher) order neighbours of the flesh color node, or not connected to it at all

To assess how our NLP model performed on an unknown corpus, we deployed it on a testing corpus of 4023 abstracts from PubMed articles. Watson retrieved a KN with a total of 681 nodes and 976 unique edges (Fig. 3a), more than in Case 1 (293 resp. 551), which means our model was able to identify new nodes and edges in this corpus. Carotenoid was again the primary centroid of this network, with 107 first-order neighbors. Our trait under study, tuber flesh color, has 21 first-order neighbors, comprising 9 genes / proteins and 12 metabolites (see Cytoscape network at [26]).

While our model is tailored toward potato tuber flesh color (ranging between white and orange), additional traits and their respective biological associations were detected as well. For example, the KN from the test set also detected genes/proteins and metabolites which influence other traits, such as enzymatic discoloration, tuber initiation, tuber development, tuber maturation, cooking types, stolon swelling, flower development etc. (Fig. 3b). This illustrates that the information content extends beyond the specific use case. Moreover, our NLP model can extract information related to tuber flesh color in a wider context than the use case only, without requiring further specific training.

#### Identifying emerging candidates with time analysis

To assess the accumulation of knowledge over time, the abstracts of the test set were organized in subsets ordered chronologically (i.e. by the date of their publication). Starting from the year 2000 and incrementing yearly (i.e. all publications up to 2000, all publications up to 2001, …, all publications up to 2016), subsets were formed. Each of these subsets was used to construct a separate KN. A network of a given year is always a subset of a KN from the following years and a superset of the previous years.

To study the development of entity connections with regard to our trait of interest (tuber flesh color), we worked backwards. The most recent collection was the most complete, so the nodes widely concerning tuber flesh color were chosen (color, flesh, flesh color, flesh trait, orange flesh color, tuber color, tuber flesh, tuber flesh color, white flesh color, yellow-orange flesh color) and are henceforth referred to as flesh color nodes. We focused our attention on the nodes that eventually ended up directly connected to a flesh color node. Then, we tracked the distance of these selected nodes to each individual flesh color node, and the changes over time. Supplementary File 2 shows an example of such a table for changes occurring between 2009 and 2010. Scripts were finally written to parse the collections for all years in the corpus. Based on these year-by-year summaries, a master summary table was made (Table 2).

**Table 2:** Overview of yearly changes in the network, based on the individual year summaries (for example Supplementary File 2). Each column represents a year, with an eventual neighbor of flesh color nodes listed in each row. The distances are the shortest path, at the time, from the node indicated to any flesh color node

node / year	2000	2001	2002	2003	2004	2005	2006	2007	2008	2009	2010	2011	2012	2013	2014	2015	2016
CCD	x	x	x	x	3	3	3	3	3	3	1	1	1	1	1	1	1
CHY	x	x	x	x	x	x	x	2	2	2	1	1	1	1	1	1	1
DXS	1	1	1	1	1	1	1	1	1	1	1	1	1	1	1	1	1
PSY	2	2	2	2	2	1	1	1	1	1	1	1	1	1	1	1	1
TP	x	x	x	x	x	x	x	x	x	3	3	2	2	2	2	2	1
abscisic acid	x	x	4	2	2	2	2	2	2	1	1	1	1	1	1	1	1
aminocyclopropane-1-carboxylic acid	x	x	x	x	x	5	5	5	5	1	1	1	1	1	1	1	1
anthocyanin	x	x	x	x	x	x	x	x	x	1	1	1	1	1	1	1	1
beta-carotene hydroxylase	x	x	4	4	4	4	4	2	2	2	1	1	1	1	1	1	1
bHLH	x	x	x	x	x	x	x	x	x	1	1	1	1	1	1	1	1
carotenoid	1	1	1	1	1	1	1	1	1	1	1	1	1	1	1	1	1
chlorophyll	x	x	x	x	x	3	3	3	1	1	1	1	1	1	1	1	1
ethylene	x	x	x	x	x	5	5	5	5	3	1	1	1	1	1	1	1
flavonoid	x	x	x	1	1	1	1	1	1	1	1	1	1	1	1	1	1
flavonol	x	x	x	x	x	x	x	x	x	x	x	x	x	x	1	1	1
hydroxycinnamic acid	x	x	x	x	x	x	x	x	1	1	1	1	1	1	1	1	1
lycopene	3	2	2	2	2	2	2	2	2	2	1	1	1	1	1	1	1
lycopene e-cyclase	x	x	x	x	x	x	2	2	2	2	1	1	1	1	1	1	1
phenolic	x	x	x	x	3	3	2	2	2	2	2	2	2	2	2	2	1
phenylalanine ammonia lyase	x	x	x	x	x	x	x	x	x	x	x	x	3	3	3	3	1
zeaxanthin epoxidase	x	x	3	3	3	3	3	2	2	2	1	1	1	1	1	1	1

Table 2 shows that the literature already contained significant indications as to the relevance of specific genes that were found to be important for potato flesh color [6]. Most prominently, both beta-carotene hydroxylase (BCH) and zeaxanthin epoxidase (ZEP) were in close proximity (2nd order neighbors) from 2007 onwards and made the transition to direct neighbors of flesh color nodes in 2010. While investigating the sentence that contributes to the transitions of ZEP in the time ranges from 2006 to 2010, we found that this gene was hypothesized to be associated with flesh color [4, 27] before experimental evidence was published in 2011. The details about the literature (publication and exact sentences) providing these connections can be found in Supplementary File 3.

Similarly, false positives such as lycopene, a metabolite not found in potato tubers, arise in the KN as first-order neighbors. While for most domain experts it is clear that lycopene is the compound responsible for flesh color in tomato, and therefore trivial to eliminate from the knowledge network as a significant player, it does reinforce the requirement for domain specialists to apply their knowledge to these results.

## Discussion

This work served as a pilot to study the benefits of using NLP platforms, such as Watson, for performing knowledge discovery in plant science literature. With the exponential increase in the number of scholarly publications and the sheer volume of available biological literature, researchers are finding it increasingly difficult to keep up-to-date with all information relevant to their field. Assembling knowledge from available literature in a single network is useful to generate new hypotheses or aid researchers in assembling a better overall picture of the components surrounding their area of interest. However, unlike for a human research expert, it is more challenging for a machine to comprehend biological insights from complex sentences and text structures of scientific literature.

The choices made in assembling our training corpus, and particularly the thematic as well as the technical preselection of articles, may have biased our model. Each NLP model has a limited scope of research questions it can address, and this particular bias functioned well enough as shown by our statistical scores. The developed type system of our NLP model cannot capture and reflect all biological complexities in knowledge networks (KNs). However, our developed NLP model is intended to only mine genotypic-phenotypic information and the underlying mechanisms from scientific literature into KNs, so that this knowledge can be structured data, easily readable by both machines and humans. The model had to learn to recognise gene, protein, metabolite and trait associations in very particular contexts precisely because of our corpus selection. Other crops, genes, proteins and traits with radically different functions and contexts may be described with different language patterns which were not present in our document set. For example, a trait like flowering time is usually described in a much different way than tissue color, and therefore to successfully capture details about, different training should be provided to the model.

Further, only generic relationships (“is related to”) of association between these entities were captured. The degree of association between two entities (positive, negative, inexplicit) was ignored in our model. The performance of our model, nevertheless, is satisfactory for the pilot study and addresses the above stated research objective. In order to optimize the efficiency of the process of manual annotation of the training set, we restricted ourselves to a limited training corpus of 34 full-text articles. Although training was thus limited, it was still sufficient to enable our model to extract similar knowledge from the test set, a collection of documents referring to different crops, traits and processes.

While making the testing corpus for our NLP model, we included literature from other *Solanaceae* crop species (tomato, capsicum, eggplant) as well. Mining and assembling information from all of these different literature resources into a single KN was somewhat controversial. Many genes and metabolites are involved in a similar bio-mechanism across these crop species. However, in some cases literature on other species may introduce noise, whereas in other cases it may be a source of ideas. There is a certain tradeoff to be observed here: the wider the scope of the processed documents, the higher the margin for noise, but also the potential. The premise for this trial, after all, was that newly published research in a broad domain of science would indiscriminately be funneled into an NLP model, to produce networks that can assist humans.

The weight function we have applied according to the number of documents that a relationship appears in can provide further insights. It is clear that, in all networks, most relationships appear only once (grey edges). However, we observe differences in the distribution of the relationships that appear more often (black edges). In Fig. 1 (full training set), there are no black edges extending outside the 5th circle region (up to 2nd order neighbours), whereas in the Fig. 3 (test set), there are black edges spanning the entire KN (region including 4th order neighbours and higher). We can hypothesise that this effect is a result of the thematic diversity of the test set, which focuses on the entire *Solanaceae* family rather than just potato. Some relationships may appear as better established because certain interactions may be investigated more in one species and not at all in another. However, there is potential in transferable knowledge between species and its visualisation in KNs in such a way. We can therefore further hypothesise that an additional extension of our scope to genera other than the *Solanaceae* (e.g. *Arabidopsis*) would yield more insights.

Although integrating more than one genus into the KNs could offer a number of benefits, as described above, we chose to refrain from doing so in this use case. Our goal was to conduct a pilot study to determine the potential of such methods and given that, it was necessary for our interpretation to select a limited domain that is relatively well-mapped. This way we can disentangle the different relationships and collect information for or research question, without a multitude of species complicating our investigation. Expert knowledge has confirmed that the limited-scope KNs distill free text into real connections between entities, and supported that the broader multi-species networks also hold promise for hypothesis generation.

A balance exists when it comes to the parts of documents that are used for text analysis. Abstracts are an easily accessible and summarized form of significant information from an article. However, different journals prescribe different formats for their abstracts and other sections of scientific articles they publish. Therefore, the quality of minable information mentioned in an abstract depends on the journal as well as the type of article. Abstracts of articles such as reviews, scientific methods, or articles that cover a wide range of topics, might not provide comprehensive minable scientific leads. For example, in the journal Nature, contributions may not always formally describe all scientific leads in their abstract, and results are more frequently mentioned in the main text.

It is worth mentioning that there were instances where the NLP approach failed to meet expectations. In cases where biological entities were abbreviated, or associations between two entities were mentioned in more than one sentences, our NLP model could not predict these entities and relationships. Watson’s type system includes facilities to co-refer abbreviated entries or pronouns to their original forms. However, due to the relatively small number of instances in our training corpus, Watson’s NLP model was not able to capture these entities and relations. However, Watson is not unique in this respect. In fact, most NLP tools suffer from the same flaw. Biological abbreviations are haphazard. Frequently, two biological concepts have the same abbreviation. For example, an abbreviation MIC might mean Minimal Inhibitory Concentration, or refer to a Major Histocompatibility Complex (MHC) class I chain related (MIC) gene. Training on a larger corpus might increase accuracy in predicting the correct entities.

Overall, our work produced a model that powered the construction and time analysis of meaningful KNs under restricted-effort conditions. We conclude that having the information we describe above available can provide key indications of scientifically relevant links, before such links are experimentally substantiated or published. The main factor that would encourage and facilitate hypothesis generation is the integration of knowledge into networks, where nodes that are not directly connected can nevertheless be close (e.g. 2nd or 3rd order neighbours). The integration perspective is also important for accumulating knowledge from multiple species into the same network, though this approach has inherent risks. All in all, we believe that a more intensive effort, for both training set size and type system definitions, would yield improved results and could play an important role in bringing together diverse information from large literature corpora and in hypothesis generation. The edges in our KNs are weighed based on the number of documents that each of them appears in.

In the future, we would like to experiment with further weight attribution methods, perhaps based on experimentally significant information from curated databases, or the number of times a particular relationship occurs in text. Cross-referencing with curated resources would serve these networks well, as experimentally verified relationships could be indicated to help filter out less reliable (negative or circumstantial) relationships in the text. As a result, text mining could be used more productively to compare established and emerging knowledge in different ways. This approach stands in contrast to others where databases establish links between their records and publications supporting them, as is the case in pubmed2ensembl BioMart [11]. Another way that our NLP model could be applied is in literature annotation on online journal articles. It could detect gene-trait associations and highlight ones that have been previously seen, and even include indications about the frequency of their occurrence. This could aid readers in understanding and appreciating the novelty of the claims presented.

## Conclusions

Our work strongly indicates that the computer-assisted extraction of knowledge from plant science literature can facilitate research. The results of our time analysis suggest that the individual components necessary for the formulation of new hypotheses may be published but remain unassociated for longer periods. Therefore, integrating these components into comprehensive knowledge networks can accelerate the generation of new hypotheses.

## Methods

### Experimental corpora

To make a supervised NLP model, we assembled scientific articles into 2 corpora, comprising a training set and a test set. The training set consisted of open source full-text articles, while the test set was built from PubMed abstracts.

The training corpus is a collection of 34 full-text scientific articles (see Supplementary File 4) which focus on tuber flesh color and known biological entities like metabolites and proteins involved in the carotenoid pathway, for example, beta-carotene hydroxylase and zeaxanthin epoxidase [7]. This corpus was the result of a search on various scholarly platforms, where we scrutinised not only the content, but also the way it was presented. The training set was manually annotated with Watson Knowledge Studio (WKS). As WKS has a limitation in detecting entity relationships only within a sentence, we eliminated articles that had no or few examples of relationship mentions in the same sentence. WKS uses these manual annotations to generate a supervised NLP model that can capture phenotypic tuber traits and the associated genes, proteins and metabolites. Later, we assessed the capabilities of this supervised NLP model to construct a knowledge network (KN) on this training set as well as on a larger test set.

The test set consists of 4023 abstracts from PubMed from the years 2000 to 2016 (which can be found at [28]). These abstracts are plant genetics-based articles which focus on 4 major Solanaceous crops (tomato, potato, eggplant and capsicum). To limit the scope of the NLP model to find direct genomic associations related to tuber flesh color, no pathogen related articles were included in the test set. Our developed NLP model is capable of extracting KNs for the tuber flesh color trait. However, the articles in the test sets deal with a variety of different topics in plant genetics and are not limited only to the tuber flesh color trait. This test set challenges the NLP model to a more real-world application, as opposed to a restricted use case in our training set.

In addition, to analyze the difference between information contained in abstracts and full text representations of an article, we divided the training set into section-based subsets. We also divided the test set of abstracts into subsets based on their year of publication, to study the evolution of knowledge over time.

### Watson knowledge studio and Watson explorer

IBM’s Watson Knowledge Studio (WKS) is a proprietary text mining solution. It can be used to build machine learning models that perform named entity recognition (NER) and relationship extraction, using state of the art methods [29–32]. The models can be tailored to different kinds of text (e.g. marketing, legal, scientific), and customized as to the type of annotations they produce.

To build a machine learning annotator in WKS, users must first define a type system to establish the “entities” (i.e. categories/classes of things that they wish for it to capture) and the “relations” between them. With the type system in place, they mark all occurrences (“mentions”) of these entities and relations in collections of representative texts, producing a ground truth. Part of these collections, the training set, is then analyzed by WKS for linguistic structures, patterns and nuances specific to the domain, to produce the machine learning model. The other part, the test set, is only used to quantify the performance of the model (precision, recall). The type system and the annotations can be changed iteratively until the model performs satisfactorily.

**Fig. 4:**
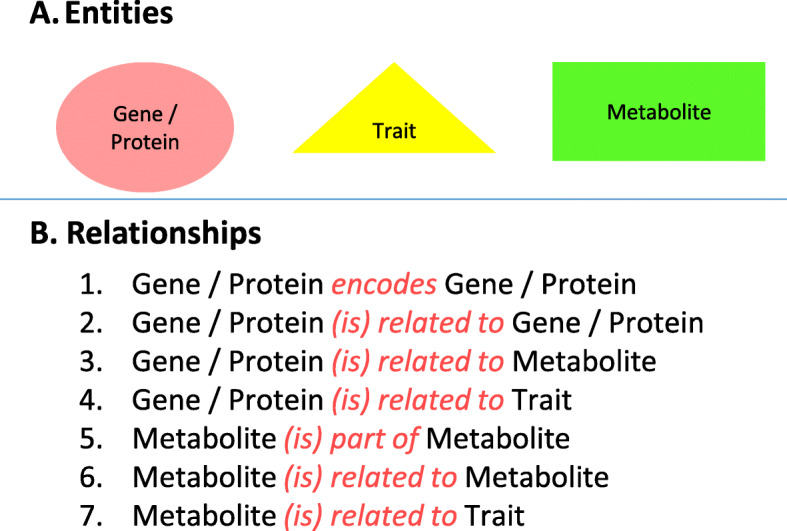
Watson Knowledge Studio (WKS) configurations of the type system for a customized NLP annotator. **a** 3 types of entities in the type system. **b** 7 types of relationships defined in the type system of an annotator

We used WKS to train a NLP model, which we then deployed on the same training set and a further test set. The final type system of our model comprised three entities (Gene/Protein, Metabolite, Trait) and seven relations between them, as seen in Fig. 4. We attained the best results with relations of a simple and all-encompassing nature, which is why many of the relations are only labeled as “related to”. The exceptions (“encodes”, “part of”) were included since the high number of instances in the corpus allowed WKS to produce models that could successfully identify them in the text.

Each entity can be supported by an entity-specific dictionary. Dictionaries are used in a pre-annotation step of NER, before the corpus is annotated manually. To minimize noise (undesirable annotation of entities and relations), all dictionaries were made small and are limited to molecular entities known to be associated with tuber flesh color or with the carotenoid pathway. We selected our preferred labels from known molecular databases or ontologies. The Gene/Protein and the Metabolite dictionaries contain 183 genes/proteins and 85 metabolites, respectively. 56 potato-related traits taken from the Solanaceae Phenotype Ontology [33] comprise the Trait dictionary.

Watson Explorer (WEx) can use the model to annotate new documents. A schematic of its pipeline can be seen in Supplementary File 5. Its outputs are text documents in XML/CAS (eXtensible Markup Language/content and structure) files, containing annotations of the entities and their relations that have been extracted, and their documents (and document position) of origin. We use these XML/CAS files to build our KNs.

### Modeling decisions

To train our NLP model to capture KNs of only genotypic-phenotypic entities and their relationships, the type system underwent a number of major changes and revisions in an iterative process. With trial-and-error optimization, entities and relationships were introduced as well as discarded, based on how well the knowledge is captured and presented in the KN. In our analysis, a knowledge triple is defined as a data structure consisting of two entities and a label for their underlying relationship.

Some modeling decisions important to be mentioned are presented below.

- Biological entities that were tested but not included in the final model:
  - biochemical processes
  - metabolic pathways
  - trait values
  - organism names, species names and genotypes
  While these biological entities occur in text and contain sources of knowledge to understand the biological mechanisms involved in the phenotypes, the numbers of mentions in the text were insufficient for WKS to adequately train a model. We therefore chose not to include these entities in the type system of our NLP model. Furthermore, including these entities in our model would have shifted the focus away from the research question of mining genotypic-phenotypic relationships in text.
- Combination of genes and proteins to a single entity:
  Initially, we kept genes and proteins as two separate entities. However, during manual annotation, difficulties were encountered in distinguishing between the two, as they are frequently used interchangeably in the text. Furthermore, for subject matter experts, there is little information lost by combining them, and separating them introduced many misclassifications. Hence, in our type system genes and proteins are a single entity.
- Annotation rule for metabolites (specific metabolite mentions vs generic mentions):
  Metabolites are included in scientific literature in different forms. Mentions may consist of specific composite terms (e.g. petunidin-3-p-coumaroyl-rutinoside-5-glucoside) or more generic ones (e.g. carotenoids). According to our type system, we annotated all forms of metabolite mentions as in this way we can capture both knowledge triples with specific entities and knowledge triples with generic entities.
- Annotation rules for genes:
  As is the case with metabolites, genes may be introduced in different formats. Sometimes the full name is presented (zeaxanthin epoxidase), sometimes the short form (ZEP), and other times there is a species indicator as a prefix (*Le*ZEP [*Lycopersicon esculentum* ZEP]). We chose to annotate all these cases to train the model.

### Building and visualization of knowledge networks

For the construction of a KN, only entities with relationships were used. The mention of an entity by itself, with no connections, was not included in the KN. With help of Python scripts, we filtered out data of entities and relationships data from XML/CAS files [34]. This script captured relationships as knowledge triples in easily parsable CSV (comma-separated values) files containing the relationship ID, relationship type, original mention of each entity, entity label, entity type, document in which this sentence occurred, sentence position and position of the source and target nodes.

As various entities appear in a variety of spellings in the corpus (e.g. *β*-carotene, b-carotene, beta-carotene), we also included a normalization step, attributing an additional preferred label to each entity. This was done manually on the list of individual entities that had been extracted. In the normalization process we first converted all spellings of entities and relationships to American English uppercase characters. Additionally, prefixes relating to species were removed from gene names. For example, the term *St*AN1, referring to anthocyanin 1 in *Solanum tuberosum* (potato), was converted to AN1. Similarly, suffixes indicating individual members of gene families were also removed, for example BCH1 and BCH2 (both referring to forms of beta-carotene hydroxylase), were converted to beta-carotene hydroxylase.

For metabolites, EC number references were converted to full names of enzymes. Further, apostrophes and # notations were removed, e.g. flavonoid-3’,5’-hydroxylase becomes flavonoid-3,5-hydroxylase, 9#-cis-neoxanthin becomes 9-cis-neoxanthin. Lastly, all abbreviations were expanded to the long form, for example, NCED2 into 9-cis-epoxycarotenoid dioxygenase. These preferred labels were based on Uniprot [35] for genes/proteins, KEGG [36] for metabolites, and the Solanaceae Phenotype Trait Ontology [37] for traits.

While the above steps reduce the specificity of a particular entity (for example we labeled BCH1 and BCH2 as BCH), as is always the case with tokenization, this simplification boosts network connectivity, despite the loss of information.

Finally, Cytoscape version 3.7.1 was used to visualize these KNs [38]. Cytoscape can plot KNs using CSV files as input. These networks also contain a weight function based on the number of documents that each edge appears in. We chose to indicate this document frequency with two colors: grey, when an edge appears only once, and black when it appears more times. This differentiation allows us to distinguish between potentially novel and more broadly investigated associations.

## Supplementary Information

**Additional file 1** Confusion matrix. A text file (.csv) containing a confusion matrix table displaying the entity detection per article for the full training set of 34 articles.

**Additional file 2** Summary table of the single-year difference in connections between flesh color and its eventual neighbours. A PDF document showing the degrees of separation between each flesh color node, and the nodes that eventually became its direct neighbours.

**Additional file 3** Tracing critical connections between zEP/BCH and flesh color. A PDF document file detailing the critical connections between BCH/ZEP and flesh color, in 2007 and 2009, as mentioned in the Results section.

**Additional file 4** List of training set documents. A text file (.csv) containing the list of 34 articles used in the training set.

**Additional file 5** Schematic of watson explorer’s pipeline. A.pdf file with a diagram showing the Statistical Information and Relation Extraction (SIRE) pipeline used by Watson.

## Data Availability

All data generated or analyzed during this study are included in this published article and its supplementary files. Additionally, the supervised NLP model made on Watson Knowledge Studio (WKS) to extract genotypic-phenotypic relations in scientific articles of potato is archived here [39]. Declarations

## Abbreviations

BCH: Beta-carotene hydroxylase
CAS: Content and structure
CHY2: Beta-carotene hydroxylase 2
CSV: Comma-separated values
FN: False negatives
FP: False positives
IBM: International Business Machines Corporation
KN: Knowledge network
*Le*ZEP: *Lycopersicon esculentum* ZEP
MHC: Major histocompatibility complex
MIC: Minimal inhibitory concentration
MIC: MHC class I chain-related
NER: Named entity recognition
NLP: Natural language processing
*St*AN1: *Solanum tuberosum* anthocyanin 1
TP: True positives
WEx: Watson Explorer
WKS: Watson Knowledge Studio
XML: eXtensible Markup Language
ZEP: Zeaxanthin epoxidase
