# Supplementary material for: Extracting knowledge networks from plant scientific literature: potato tuber flesh color as an exemplary trait
Source: BMC Plant Biol. 2021 Apr 24;21:198. doi: 10.1186/s12870-021-02943-5 (PMC8070292; doi:10.1186/s12870-021-02943-5)
Supplement: Supplementary file 2 — Additional file 2 Summary table of the single-year difference in connections between flesh color and its eventual neighbours. A PDF document showing the degrees of separation between each flesh color node, and the nodes that eventually became its direct neighbours. [file 12870_2021_2943_MOESM2_ESM.pdf]

Additional file 2 – Summary table of the single-year difference in connections between flesh color and its eventual neighbours

2009→2010		flesh color-like nodes						min
		flesh	flesh color	tuber flesh	tuber flesh color	white flesh color	yellow-orange color	
eventual direct neighbours to flesh color-like nodes	**CCD**	3→1	x→3	6→3	x→3	x→1	x→2	3→1
	**CHY**	2	x→1	5→3	x→2	x→3	x→3	2→1
	**DXS**	1	x→3	5→3	x→3	x→3	x→3	1
	**PSY**	1	x→3	5→3	x→3	x→3	x→2	1
	**TP**	3	x→5	7→4	x→5	x→4	x→4	3
	**abscisic acid**	1	x→3	5→2	x→3	x→2	x→3	1
	**aminocyclopropane-1-carboxylic acid**	1	x→4	5→4	x→4	x→3	x→3	1
	**anthocyanin**	3	x→4	1	x→5	x→5	x→5	1
	**b-carotene hydroxylase**	2	x→1	5→3	x→1	x→3	x→3	2→1
	**bHLH**	5→4	x→4	1	x→5	x→5	x→5	1
	**carotenoid**	1	x→2	4→2	x→2	x→3	x→2	1
	**chlorophyll**	1	x→3	5→3	x→3	x→3	x→3	1
	**ethylene**	3	x→5	7→5	x→5	x→4	x→1	3→1
	**flavonoid**	1	x→3	3	x→3	x→3	x→3	1
	**flavonol**	x	x	x	x	x	x	
	**hydroxycinnamic acid**	1	x→4	5→4	x→4	x→3	x→4	1
	**lycopene**	2	x→3	5→3	x→3	x→2	x→1	2→1
	**lycopene e-cyclase**	2	x→1	5→2	x→3	x→3	x→3	2→1
	**phenolic**	2	x→3	4→3	x→3	x→4	x→3	2
	**phenylalanine ammonia lyase**	x	x	x	x	x	x	
	**zeaxanthin epoxidase**	2	x→2	5→1	x→3	x→3	x→3	2→1
