# Supplementary material for: Extracting knowledge networks from plant scientific literature: potato tuber flesh color as an exemplary trait
Source: BMC Plant Biol. 2021 Apr 24;21:198. doi: 10.1186/s12870-021-02943-5 (PMC8070292; doi:10.1186/s12870-021-02943-5)
Supplement: Supplementary file 3 — Additional file 3 Tracing critical connections between zEP/BCH and flesh color. A PDF document file detailing the critical connections between BCH/ZEP and flesh color, in 2007 and 2009, as mentioned in the Results section. [file 12870_2021_2943_MOESM3_ESM.pdf]

Additional file 3 – Tracing critical connections between ZEP/BCH and flesh color

Based on Table 2, this section elaborates on the specific source of the connections between ZEP, BCH and color nodes.

In 2007, Diretto et al. [1] wrote:

- "*Silencing of beta-carotene hydroxylase increases total carotenoid and beta-carotene levels in potato tubers*" (paper title)

In this case, a new connection has been drawn between b-carotene hydroxylase and carotenoid; carotenoid has been a long time neighbour of flesh.

- "*Changes in endogenous gene expression were extensive and partially overlapping with those of LCY-e silenced tubers: CrtISO, LCY-b and ZEP were induced in both cases, indicating that they may respond to the balance between individual carotenoid species.*"

Here a new connection is drawn between ZEP and carotenoid.

In 2010, similarly, Kloosterman et al. [2] and Wolters et al. [3] stated, respectively:

- "*Elevated expression level of a dominant allele of the beta-carotene hydroxylase (bch) gene was associated with yellow flesh color through mapping of the gene under a major QTL for flesh color on chromosome 3.*" and "*The identified candidate genes for tuber flesh color (bch) and cooking type (tlrp) can provide useful markers for breeding schemes in the future.*"
- "*We observed that among eleven beta-carotene hydroxylase 2 (Chy2) alleles only one dominant allele has a major effect, changing white into yellow flesh colour*", and "*Analysis of zeaxanthin epoxidase (Zep) alleles showed that all (diploid) genotypes with orange tuber flesh were homozygous for one specific Zep allele.*"

The above sentences enabled Watson to extract direct relationships from ZEP and BCH references to flesh color.

References

[1] G. Diretto *et al.*, "Silencing of beta-carotene hydroxylase increases total carotenoid and beta-carotene levels in potato tubers," *BMC Plant Biology*, vol. 7, 2007.

[2] B. Kloosterman *et al.*, "From QTL to candidate gene: genetical genomics of simple and complex traits in potato using a pooling strategy.," *BMC genomics*, vol. 11, p. 158, 2010.

[3] A. M. A. Wolters, J. G. A. M. L. Uitdewilligen, B. A. Kloosterman, R. C. B. Hutten, R. G. F. Visser, and H. J. van Eck, "Identification of alleles of carotenoid pathway genes important for zeaxanthin accumulation in potato tubers," *Plant Molecular Biology*, vol. 73, no. 6, pp. 659–671, 2010.
