# Supplementary material for: Extracting knowledge networks from plant scientific literature: potato tuber flesh color as an exemplary trait
Source: BMC Plant Biol. 2021 Apr 24;21:198. doi: 10.1186/s12870-021-02943-5 (PMC8070292; doi:10.1186/s12870-021-02943-5)
Supplement: Supplementary file 5 — Additional file 5 Schematic of watson explorer’s pipeline. A.pdf file with a diagram showing the Statistical Information and Relation Extraction (SIRE) pipeline used by Watson. [file 12870_2021_2943_MOESM5_ESM.pdf]

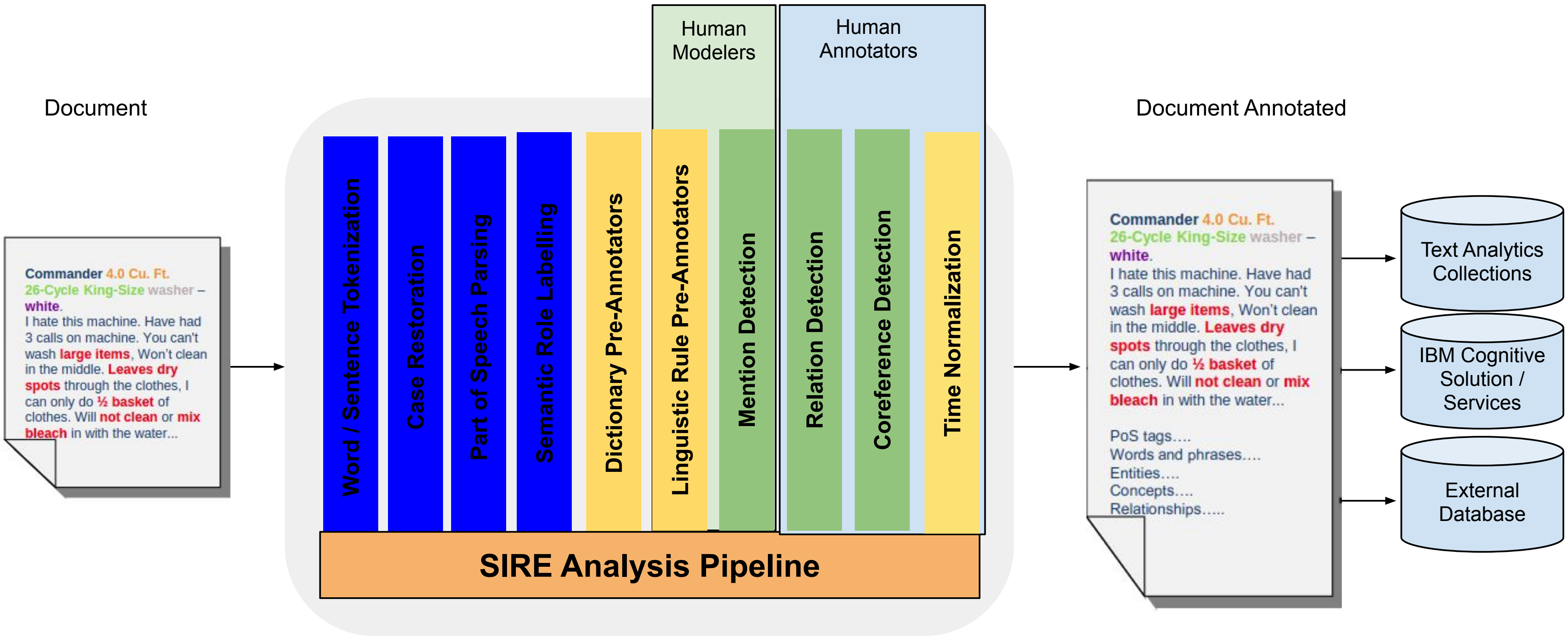

Additional File 5: A schematic representation of the Statistical Information and Relation Extraction (SIRE) pipeline used by Watson.
